# Bilateral thalamic encephalitis due to Epstein–Barr virus in an elderly patient: a case report and literature review

**DOI:** 10.1186/s12883-025-04563-0

**Published:** 2025-12-01

**Authors:** Francesco Perrotta, Federica Vitale, Ludovica De Marco, Andrea Sparascio, Pierluigi Morciano, Francesca De Salvo, Gaetano Iosa, Daniela Rizzo, Donato Piscopiello, Daniele Gemma

**Affiliations:** 1 Department of Anesthesia and Intensive Care, Azienda Ospedaliera “Cardinale G. Panico”, Tricase (LE), Italy; 2 Department of Anesthesiology and Intensive Care, Università Campus Bio-Medico Di Roma, Rome, Italy

**Keywords:** Epstein–Barr virus, Encephalitis, Thalamus, Diffusion-weighted imaging, ADC, Elderly, Case report

## Abstract

Bilateral thalamic encephalitis due to Epstein–Barr virus (EBV) is a rare and severe manifestation of EBV infection, often leading to rapid neurological deterioration and poor outcomes. We report the case of an 82-year-old man with a history of arterial hypertension and a previous herpes zoster infection who presented to the emergency department with high fever and acute neurological impairment evolving into coma (Glasgow Coma Scale, GCS 3). Cerebrospinal fluid (CSF) analysis revealed mild lymphocytic pleocytosis, elevated protein and normal glucose. EBV-DNA was detected by polymerase chain reaction (PCR) and serology confirmed positive anti-EBV IgM antibodies. Autoimmune and paraneoplastic panels, including anti-NMDA receptor, anti-GQ1b, anti-MOG, anti-AQP4 and anti-LGI1 antibodies, were negative. Magnetic resonance imaging (MRI) demonstrated bilateral thalamic hyperintensities on T2-weighted and FLAIR sequences, with restricted diffusion on DWI and corresponding hypointensity on ADC maps, without contrast enhancement. Angiography showed normal patency of the main intracranial vessels. Differential diagnoses such as autoimmune encephalitis, acute disseminated encephalomyelitis, Bickerstaff’s brainstem encephalitis, vascular, metabolic and other viral causes were excluded. Despite prompt antiviral and corticosteroid therapy and intensive supportive management, the clinical outcome was poor. A comprehensive literature review was performed, highlighting key clinical and radiological findings from previously published cases of EBV-related bilateral thalamic encephalitis, to improve recognition and understanding of this rare and devastating condition.

## Introduction

Epstein–Barr virus (EBV), a member of the Herpesviridae family, is a widespread pathogen that infects over 90% of adults worldwide and is typically associated with infectious mononucleosis. Neurological complications such as meningitis, encephalitis, cerebellitis, transverse myelitis and polyradiculitis are uncommon, accounting for less than 1% of all EBV infections [1–3].

Among these, EBV-associated encephalitis is rare and presents with a broad clinical spectrum, from mild confusion to deep coma, particularly in elderly or immunocompromised patients [4, 5].

Bilateral thalamic involvement due to EBV is exceptionally rare and represents a diagnostic challenge because this imaging pattern overlaps with several other neurological disorders, including acute disseminated encephalomyelitis (ADEM), Bickerstaff’s brainstem encephalitis, autoimmune encephalitis such as anti-NMDA receptor encephalitis, viral encephalitides of different aetiology, metabolic disorders (such as Wernicke’s encephalopathy), and vascular causes such as deep cerebral venous thrombosis [6–8].

Differentiating these entities is critical, as they require distinct therapeutic approaches and have markedly different prognoses.

Early recognition of EBV-related encephalitis is essential to initiate prompt antiviral and supportive therapy, although specific treatment efficacy remains uncertain. In this report, we describe an elderly patient who developed acute bilateral thalamic EBV encephalitis confirmed by positive EBV-DNA in the CSF and characteristic MRI findings.

We also provide a brief review of the literature on previously published cases to highlight diagnostic challenges and current understanding of the pathophysiology, imaging features and outcomes associated with EBV encephalitis involving the thalami.

## Case report

An 82-year-old man with a history of arterial hypertension and a previous episode of herpes zoster infection affecting a thoracic dermatome two years earlier, completely resolved without sequelae, was admitted to the emergency department with high fever (38.7 °C), confusion and reduced responsiveness that had progressively worsened over the previous 24 h.

He had no history of recent vaccinations, respiratory or gastrointestinal prodromal symptoms, or travel abroad. There was no known exposure to infectious diseases.

On admission, the patient appeared somnolent but arousable to painful stimuli, disoriented and unable to follow commands. Pupils were isochoric and reactive to light, and cranial nerve examination was unremarkable. Muscle tone was diffusely reduced, deep tendon reflexes were decreased, and plantar responses were bilaterally extensor. There were no signs of meningeal irritation. Cardiovascular, pulmonary and abdominal examinations were normal.

Initial laboratory tests revealed leukocytosis (12.4 × 10⁹/L) and elevated C-reactive protein (CRP) (8.6 mg/dL), while renal and hepatic function tests were within the reference range (Table 1). Electrolyte balance and blood glucose levels were normal.

**Table 1:** Baseline laboratory investigations on admission

Parameter	Result	Reference range
White blood cells	12.4 × 10⁹/L	4.0–10.0 × 10⁹/L
CRP (mg/dL)	8.6	< 0.5
Creatinine (mg/dL)	1.0	0.7–1.3
ALT (U/L)	28	< 45
AST (U/L)	30	< 45
Sodium (mmol/L)	139	136–145
Potassium (mmol/L)	4.2	3.5–5.0
Glucose (mg/dL)	96	70–110

Given the presence of fever and inflammatory markers, a urinary tract infection was suspected, and empirical antibiotic therapy with piperacillin–tazobactam was initiated.

Despite treatment, the patient’s level of consciousness rapidly deteriorated within the next 24 h, reaching a Glasgow Coma Scale (GCS) score of 3 (E1V1M1). He was therefore transferred to the intensive care unit (ICU), where he required endotracheal intubation and mechanical ventilation.

Cerebrospinal fluid (CSF) analysis revealed mild lymphocytic pleocytosis (25 cells/mm^3^), elevated protein (112 mg/dL) and normal glucose (64 mg/dL). EBV-DNA was detected by polymerase chain reaction (PCR), whereas PCR for HSV-1/2, varicella-zoster virus (VZV), cytomegalovirus (CMV), enteroviruses and JC virus were all negative (Table 2).

**Table 2:** Cerebrospinal fluid (CSF) analysis and microbiology

Parameter	Result	Reference range
White blood cells (cells/mm^3^)	25 (lymphocytic)	0–5
Protein (mg/dL)	112	15–45
Glucose (mg/dL)	64	40–70
EBV-DNA PCR	Positive	Negative
HSV-1/2 PCR	Negative	Negative
VZV PCR	Negative	Negative
CMV PCR	Negative	Negative
Enterovirus PCR	Negative	Negative
Anti-EBV IgM	Positive	Negative
Anti-EBV IgG	Negative	Negative

Serological testing confirmed anti-EBV IgM positivity with negative anti-EBV IgG, supporting a recent primary infection.

A complete autoimmune and paraneoplastic panel was performed on both serum and CSF, yielding negative results for anti-NMDA receptor, anti-MOG, anti-GQ1b, anti-AQP4, LGI1 and CASPR2 antibodies. Paraneoplastic antibodies (anti-Hu, anti-Yo, anti-Ri, anti-Ma2 and anti-amphiphysin) were also negative.

No neoplastic lesions were detected on whole-body computed tomography. Blood and urine cultures remained sterile.

**Fig. 1:**
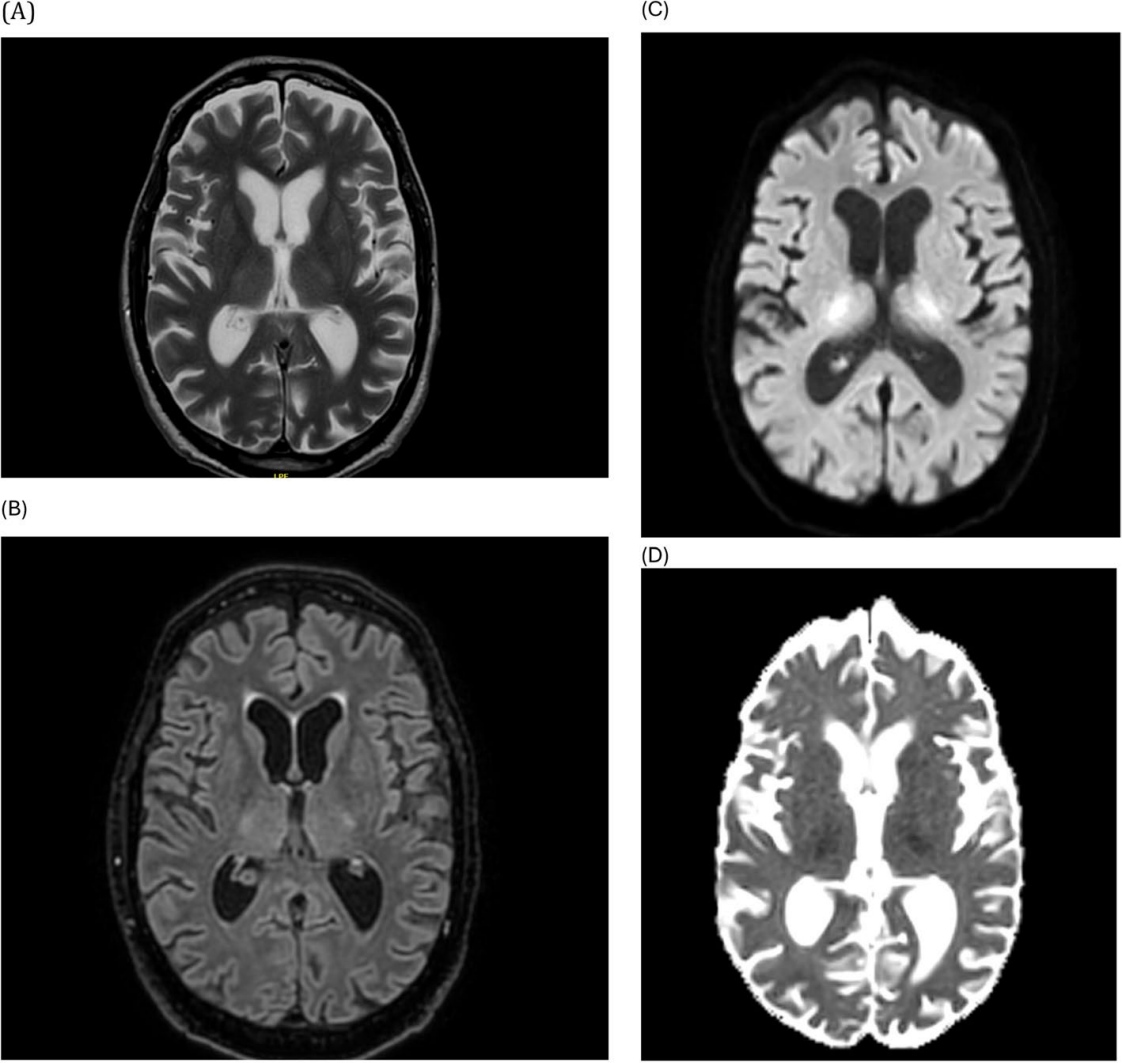
Brain MRI at presentation showing bilateral thalamic lesions: axial T2-weighted (**A**), FLAIR (**B**), DWI (**C**) and corresponding ADC map (**D**). Lesions demonstrate diffusion restriction with ADC hypointensity and no contrast enhancement

**Fig. 2:**
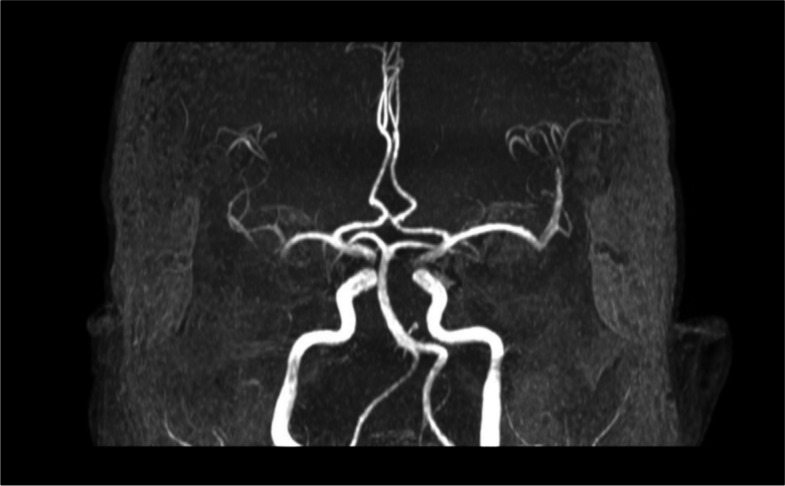
Magnetic resonance angiography demonstrating normal patency of the main intracranial arteries without occlusion or vasculitic changes

Brain magnetic resonance imaging (MRI) demonstrated bilateral thalamic hyperintensities on T2-weighted and FLAIR sequences, with restricted diffusion on DWI and corresponding hypointensity on ADC maps, consistent with cytotoxic oedema. No contrast enhancement was observed. Magnetic resonance angiography (MRA) revealed normal patency of the main intracranial vessels, ruling out both arterial and venous occlusion as well as vasculitic changes (Figs. 1 and 2).

A follow-up MRI performed 10 days later showed persistent bilateral thalamic lesions with no new abnormalities.

Given the suspicion of viral encephalitis, intravenous aciclovir (10 mg/kg every 8 h) was promptly initiated, together with high-dose corticosteroids (methylprednisolone 1 g/day for 5 days) and broad-spectrum antibiotics.

Supportive therapy included airway management, vasopressor support and nutritional assistance.

Despite aggressive treatment, the patient remained comatose and exhibited no neurological improvement. On day 18 of hospitalisation, he died of multi-organ failure.

## Discussion

Epstein–Barr virus (EBV) encephalitis is a rare but severe neurological complication that may affect both immunocompetent and immunocompromised patients. The pathogenesis remains unclear and likely involves direct viral invasion as well as post-infectious immune-mediated mechanisms. The most frequent MRI findings include multifocal cortical and subcortical lesions, basal ganglia and brainstem involvement; however, bilateral thalamic lesions are exceedingly rare and have been reported only in a small number of cases in the literature [1–3].

In our patient, EBV-DNA positivity in the cerebrospinal fluid (CSF) confirmed the viral etiology, while the absence of other neurotropic viruses and negative autoimmune and paraneoplastic panels excluded alternative diagnoses such as acute disseminated encephalomyelitis (ADEM), Bickerstaff’s brainstem encephalitis, and anti-NMDA receptor autoimmune encephalitis.

This is consistent with previous reports suggesting that, although EBV-PCR in CSF may occasionally be non-specific, high viral copy number and congruent clinical-imaging findings strongly support a causal role [4, 5].

Differential diagnosis of bilateral thalamic involvement is challenging. Besides viral encephalitis, the main differentials include deep cerebral venous thrombosis, Wernicke’s encephalopathy, metabolic or toxic encephalopathy, and Creutzfeldt–Jakob disease. In this case, the absence of vascular occlusion on MRA and the typical DWI/ADC pattern of cytotoxic oedema were crucial to exclude ischemic or venous causes and to support an inflammatory/infectious process.

The clinical evolution was rapidly progressive, with deep coma within 24 h, a feature described in the most severe forms of EBV encephalitis.

A review of the literature identified fewer than 25 cases of EBV encephalitis with bilateral thalamic involvementpublished between 1980 and 2024 (Table 3). These reports show a high mortality rate (> 40%), particularly in elderly patients, and poor neurological outcomes in survivors despite antiviral therapy [6–8].

**Table 3:** Reported cases of EBV encephalitis with bilateral thalamic involvement (1980–2024)

Author, Year	Age/Sex	Immune status	MRI findings	CSF EBV-PCR	Treatment	Outcome
Portegies et al., 2000 [3, 9]	47/M	Immunocompetent	Bilateral thalami + midbrain	Positive	Acyclovir + steroids	Recovery with mild deficits
Sener RN, 2003 [4, 10]	10/F	Immunocompetent	Bilateral thalami (DWI restriction)	Positive	Acyclovir	Full recovery
Salgado RA et al., 2015 [5, 11]	63/M	Immunocompetent	Thalami + basal ganglia	Positive	Acyclovir + steroids	Death
Guan J et al., 2022 [8, 12]	71/F	Immunocompetent	Bilateral thalami (FLAIR/DWI)	Positive	Acyclovir + IVIG	Partial recovery
Present case, 2025	82/M	Immunocompetent	Bilateral thalami (T2/FLAIR + DWI/ADC)	Positive	Acyclovir + steroids	Death

Table 3 summarises representative cases, including clinical features, MRI findings, and outcomes (adapted and updated from the previously reported series).

The optimal therapeutic approach remains uncertain. Acyclovir is generally recommended empirically for suspected viral encephalitis, even though EBV is less sensitive to it in vitro. Some studies suggest a potential benefit of ganciclovir or foscarnet, but clinical data are limited.

Adjunctive high-dose corticosteroid therapy is commonly administered in severe cases to attenuate the inflammatory and immune-mediated component. In our case, despite combined antiviral and steroid therapy, the outcome was fatal, highlighting the dismal prognosis in elderly patients with diffuse bilateral thalamic involvement.

The mechanisms underlying EBV-related thalamic injury remain speculative. Direct viral invasion of neurons and glial cells, secondary immune-mediated demyelination, and microvascular inflammation have all been proposed [13–15]. Post-mortem histopathological studies show perivascular lymphocytic infiltration and microglial activation, supporting a dual mechanism of injury.

The present case adds to the growing evidence that EBV encephalitis may present with stereotyped bilateral thalamic lesions even in immunocompetent individuals, and that early neuroimaging and molecular CSF testing are fundamental for diagnosis.

Furthermore, our review highlights the need for standardised therapeutic protocols and prospective studies to better define the role of antivirals and immunomodulatory therapy in this rare but severe condition.

## Conclusion

EBV encephalitis with bilateral thalamic involvement is an extremely rare and life-threatening condition that requires a high index of suspicion, especially in elderly or immunocompetent patients presenting with rapidly progressive altered consciousness.

Early recognition through combined clinical, radiological and molecular evaluation is crucial for diagnosis and timely initiation of antiviral and immunomodulatory therapy, although the prognosis remains poor in most cases.

This case underscores the importance of multidisciplinary collaboration among neurologists, infectious disease specialists, intensivists and neuroradiologists for rapid differential diagnosis and management.

A systematic review of similar cases suggests that bilateral thalamic lesions should not immediately be attributed to vascular or metabolic causes, as infectious or autoimmune etiologies such as EBV may present with a comparable pattern.

Further studies are needed to elucidate the exact pathophysiological mechanisms and to establish evidence-based treatment protocols aimed at improving outcomes in this rare but severe form of encephalitis.

## Data Availability

All data supporting the findings are included in the article and its tables/figures; additional details are available from the corresponding author upon reasonable request.
